# Genetic Determinants of Clarithromycin and Fluoroquinolones Resistance in *Helicobacter pylori* in Serbia

**DOI:** 10.3390/antibiotics13100933

**Published:** 2024-10-01

**Authors:** Dusan Kekic, Milos Jovicevic, Jovana Kabic, Iva Lolic, Ina Gajic, Stefan Stojkovic, Lazar Ranin, Tomica Milosavljevic, Natasa Opavski, Ivan Rankovic, Vladimir Milivojevic

**Affiliations:** 1 Institute for Microbiology and Immunology, Faculty of Medicine, University of Belgrade, 11 000 Belgrade, Serbia; 2 Emergency Center, University Clinical Center of Serbia, 11 000 Belgrade, Serbia; 3 Clinic for Gastroenterology and Hepatology, University Clinical Centre of Serbia, 11 000 Belgrade, Serbia; 4 Euromedic, General Hospital, 11 000 Belgrade, Serbia; 5 Department of Gastroenterology and Liver Care, Royal Cornwall Hospitals NHS Trust University of Exeter, Treliske, Truro, Cornwall TR1 3LQ, UK; 6 Faculty of Medicine, University of Belgrade, 11 000 Belgrade, Serbia

**Keywords:** *Helicobacter pylori*, antibiotic resistance, clarithromycin, fluoroquinolones, genetic determinants, Serbia

## Abstract

Background/Objectives: Stomach infections by *Helicobacter pylori* can cause acute or chronic gastritis, peptic ulcers, and gastric cancer. The rise in antibiotic resistance is a significant health issue highlighted by the World Health Organization. The increasing number of treatment failures underscores the necessity for antibiotic susceptibility testing (AST). The study aimed to investigate the current prevalence and resistance to fluoroquinolones and clarithromycin with their detected mutations. Methods: Stomach biopsies from symptomatic patients were subjected to molecular testing by GenoType Helico DR kit (Hain Lifescience GmbH, Nehren, Germany). Results: Positive findings on the presence of *H. pylori* were detected in 42.4% of symptomatic patients, with the significant majority of patients (69%) having previously failed treatments. The resistance rates to fluoroquinolones and clarithromycin were 53.9% and 58.5%, respectively, with significantly higher rates in secondary resistant strains. The main resistance markers in fluoroquinolones and clarithromycin were N87K (27.4%) and A2147G (78.6%), respectively. Hetero-resistance or mixed genotypes were detected in over 20% of tested patients. During the study period, a significant increase in trends in both fluoroquinolones and clarithromycin resistance rates was observed. Conclusions: Results indicate the need for the implementation of the latest Maastricht VI Consensus recommendations for both AST whenever possible and the use of tailored guided therapy options due to high resistance rates and possible treatment failures. The GenoType Helico DR kit is a useful tool for AST, especially in cases of mixed *H. pylori* genotypes.

## 1. Introduction

*Helicobacter pylori* (*H. pylori*) is a gram-negative, flagellated, microaerophilic, curved bacterium that may colonize the human stomach. While the majority of the population (>80%) may stay asymptomatic, its chronic infection and persistent colonization are primarily associated with gastrointestinal illnesses [1,2]. Its prevalence is worldwide and affects half of the world’s population, with prevalence ranging from 35% in developed countries to a high prevalence of 90% in some developing countries [3]. Symptomatic patients and asymptomatic persons may develop various digestive diseases, such as chronic gastritis, atrophic gastritis to intestinal metaplasia, non-ulcer dyspepsia, duodenal and gastric ulcer, gastric adenocarcinoma, and gastric mucosa-associated lymphoid tissue lymphoma (MALT) [1].

Due to a paradigm shift regarding host-bacterium interaction, indication for treatment is no longer reserved for patients with clinical manifestations of infection [4]. Treatment of *H. pylori* infection is based on multiple antibiotic regimens, which are mainly empirically tailed, like standard triple therapy consisting of proton pump inhibitor (PPI) with two antibiotics—clarithromycin with either amoxicillin or nitroimidazole (metronidazole or tinidazole) or sequential therapy [4,5,6]. Eradication failures, antimicrobial resistance of *H. pylori,* and patient reinfections have been detected globally as major public health problems [7]. According to the World Health Organisation (WHO), *H. pylori* is listed as one of the 16 antibiotic-resistant bacteria that are the greatest threat to human health. It is classified in the second WHO priority group as a high-priority bacteria, with vancomycin-resistant *Enterococcus faecium,* and vancomycin-intermediate or resistant and methicillin-resistant *Staphylococcus aureus* [8]. The WHO recently updated its Bacterial Priority Pathogens List in 2024, where despite its rising resistance rates, clarithromycin-resistant *H. pylori* was removed based on evidence and expert consensus [9].

As *H. pylori* resistance rates are rising worldwide, the latest guidelines recommend that it is reasonable to perform susceptibility testing (phenotypic or genotypic), even before prescribing first-line treatment, concerning antibiotic stewardship [4]. Current data confirm a strong correlation between macrolide and quinolone use in the community and corresponding *H. pylori* resistance in Europe. Hence, in most European countries, *H. pylori* treatment with clarithromycin and levofloxacin protocols should not be prescribed without susceptibility testing [10]. Resistance of *H. pylori* to antibiotics is increasing worldwide, which has a significant effect on treatment success [11]. The eradication rates of primary therapy reported in Africa dropped from over 85% reported in 2000 to around 60% in 2020 [12]. Regional surveillance and resistance data are required to use appropriate eradication treatment locally [4]. The resistance rate progressively increased in correlation with the number of eradication attempts. As compared to patients who had only one failed therapy, those with two failures had significantly higher resistance rates [13]. Also, the misuse and overuse of macrolides highly affect antibiotic resistance rates and eradication efficacy [14].

The *H. pylori*’s resistance to antibiotics is most often due to chromosomally encoded mutations. The resistance to fluoroquinolones in *H. pylori* is mainly mediated by point mutations in the *gyrA* gene, encoding A subunit of the DNA gyrase, primarily at codons 87 (N87L, I, A or K) and 91 (D91G, N, A, Y or H) [15]. Clarithromycin resistance is, in the majority of cases (>90%), expressed by point mutations in domain V of 23S rRNA at nucleotide positions A2146 and A2147 (previously marked as A2142 and A2143) [16,17]. Less frequent mutations are also reported for clarithromycin resistance A2115G, G2141A, G2144T, T2117C, and T2289C, low-level resistance for T2182C, C2611A, C2694A, and T2717C, while others (A1821G, G1826A, T1830C, C2245T, T2289C, and G2224A) need to be yet determined [18,19,20,21,22].

In Serbia, there is a limited amount of information regarding antibiotic resistance and treatment success for *H. pylori* infection [23]. Therefore, this study aimed to summarize the first results of *H. pylori* molecular diagnostic testing from bioptic material among symptomatic patients, its resistance rates, and determinants to clarithromycin and fluoroquinolones among patient samples in Serbia.

## 2. Results

### 2.1. Detection of Helicobacter pylori

During the study period, a total of 415 patients with dyspeptic symptoms were sampled by stomach biopsy for the presence of *H. pylori*. Results of testing patient biopsy samples with the GenoType Helico DR kit showed that 176 (42.4%) samples were found to be positive for *H. pylori.*

The average age of patients in the entire population was 53.8 ± 14.9, while in the *H. pylori*-positive patients it was 50.8 ± 14.8. The female gender was more represented in the entire sample with 262 (63.1%) patients and among the *H. pylori*-positive patients with 109 (60.9%). In comparison, there were 153 (36.8%) men in the entire sample, and 70 (39.1%) among the *H. pylori*-positive. No significant gender difference was observed between *H. pylori*-positive and negative patients (*p* = 0.41).

As for the *H. pylori*-positive patients, there was a significant difference between the number of previous naïve patients (31%) and patients with previous unsuccessful eradications (69%; *p* = 0.047).

### 2.2. Detection of Antibiotic Resistance Genes

**Figure 1:**
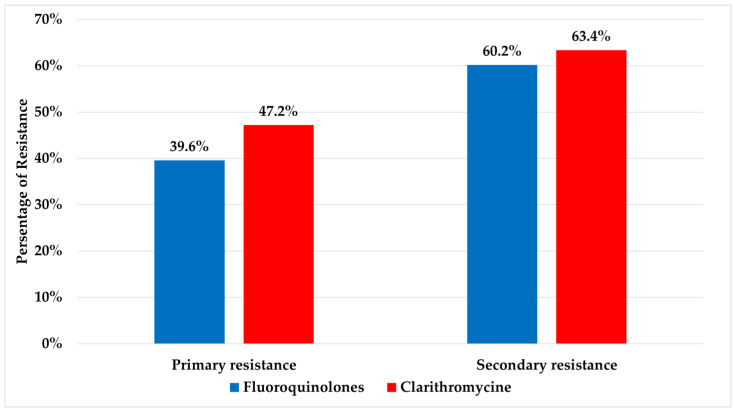
Values of primary and secondary resistance rates to fluoroquinolones and clarithromycin.

A cumulative resistance rate detected for fluoroquinolones and clarithromycin were 53.9% and 58.5%, respectively. Significant differences were detected for both fluoroquinolones and clarithromycin between primary (resistance in patients with no prior history of eradication treatment) and secondary resistant isolates (resistance in patients who have undergone eradication treatment) (*p* ≤ 0.05) and are presented in Figure 1. The relations between age and gender with resistance to fluoroquinolones and clarithromycin are presented in Table 1.

The detected mutations in fluoroquinolones resistant strains showed the highest number being found in the *gyrA* gene, on position 87 (N87K; 27.4%), followed by position 91 (D91N; 24.2%). The most commonly detected mutation in clarithromycin-resistant strains was on the *rrl* gene A2147G (78.6%). All detected mutations are presented in Table 2. Among all *H. pylori*-positive patients, 54.7% of strains were found to be resistant to both fluoroquinolones and clarithromycin. A similar distribution of dual resistance and resistance to fluoroquinolones and clarithromycin in *H. pylori*-positive patients was found between primary and secondary resistance (53.3% vs. 55.1%; 16.7% vs. 20.4%; 30.0% vs. 24.5%, respectively).

Mixed genotypes were found in 20.3% of *H. pylori*-positive patients, with more than one band of wild type and/or mutation found on a test strip. Detected mutations in these patients are presented in Table 3.

**Figure 2:**
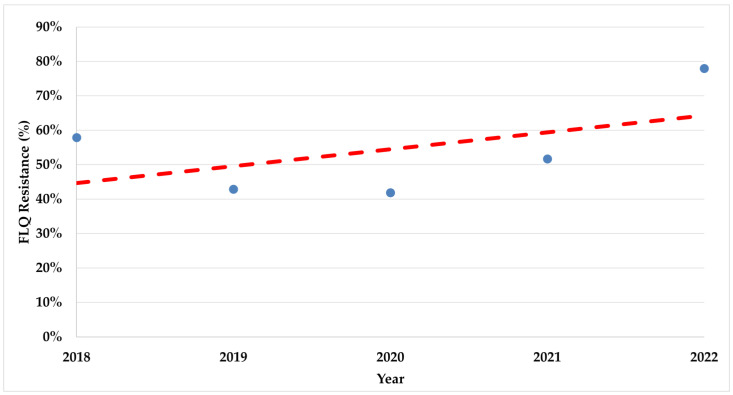
The trend of fluoroquinolone resistance rates during the study period. FLQ—Fluoroquinolones.

**Figure 3:**
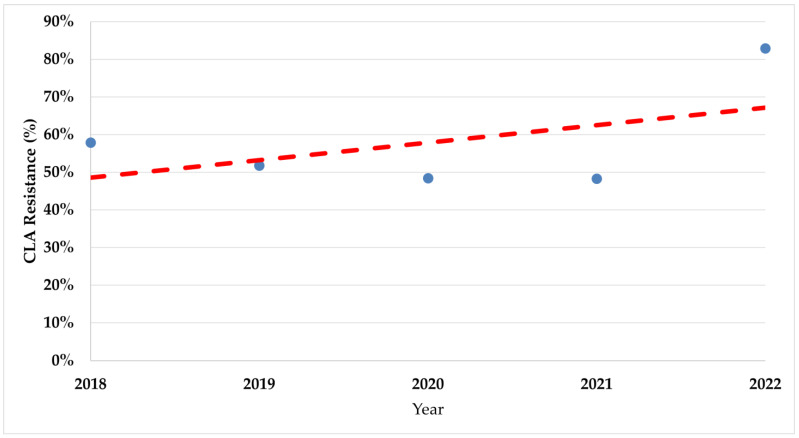
The trend of clarithromycin resistance rates during the study period. CLA—Clarithromycin.

During the study period, there was a significant increase in trends of resistance rates in both fluoroquinolones (*p* < 0.008) and clarithromycin (*p* < 0.017) (Figure 2 and Figure 3).

## 3. Discussion

The results of this study indicate that the prevalence of *H. pylori* among symptomatic patients was 42.4%, which is close to the actual global results that presented a decline among adults from 50–55% to 43% during 2014–2020 [24]. Comparing the prevalence of *H. pylori* presence among age and gender patient groups, a significant association was found among women, which is similar to that reported in an Israeli study [25].

The majority of *H. pylori*-positive patients (70%) in the study population had previous unsuccessful eradication attempts. Among the known factors yielding the unsuccessful eradication outcomes are the bacteria’s antimicrobial resistance to one or co-resistance to both given antibiotics and the presence of heterogeneity in the *H. pylori* community. In the current study, high resistance rates were detected for both fluoroquinolones and clarithromycin, which is higher than the previously published preliminary data from Serbia [23]. Data from authors in Spain show even higher rates (59% and 84%, respectively), genotypically performed, as in this study [26]. Resistance data from Southeastern Europe revealed different and controversial data, ranging from 14.3% to clarithromycin in the naïve group and very low levofloxacin resistance in both groups in Slovenia [27], to higher rates in Croatia of 34.6% in 2018 [9] to clarithromycin, and in Bosnia, which is showing an increase in levofloxacin resistance of 37% overall. Additionally, over half of the patients in this study carried strains with dual resistance to both fluoroquinolones and clarithromycin. Both high resistance rates and co-resistance of fluoroquinolones and clarithromycin are found to be a considerable cause of the decrease in the eradication rates of *H. pylori*, especially in triple therapy and levofloxacin-based regimens [28,29]. The increasing global rates of *H. pylori* resistance in treatment-naïve patients can be related to uncontrolled use of antibiotics that are usually used in *H. pylori* empirical therapy and in therapy for other frequent infections in the general population (respiratory, urine infection, COVID-19 pandemic, etc.) [30]. Overall macrolide usage increased by 19% and fluoroquinolone consumption by 64% in the period from 2000 to 2010 [31]. Also, levofloxacin resistance plays an important role in potential treatment failure [32]. Moreover, in the study population, there were detected significantly higher rates of resistance to both fluoroquinolones and clarithromycin in secondary resistant strains compared to the tested strains from naïve patients. Similar findings were reported by Greek authors, questioning the efficacy of an empirical second-line levofloxacin regimen and pointing out the need for antibiotic susceptibility-tailored therapy [33].

The 87 amino acid mutation is suggested to be more efficient than that in amino acid 91, and furthermore, their combinations are even more efficacious than those detected alone [34]. Besides being predominant, the N87K mutation was associated with higher minimal inhibitory concentration values to levofloxacin [35]. As for clarithromycin resistance, the dominance of the A2147G mutation was detected, which is in line with previous studies [13,31,36,37]. The particular mutation was previously associated with a higher risk of eradication failures [21,38], and the majority of tested patients in the current study are in this group.

Similar detection of around 20% of patients with hetero-resistant genotypes for tested antibiotics was found by other authors [13,31]. These results may also lead to therapy failure [39], as a coexistence of the wild-type and mutant strains in a patient may present dominance of the wild-type and a susceptible phenotype [34]. Also, the findings of more than one strain of *H. pylori* in patients are associated with a more severe form of the disease [40]. *H. pylori* has a possibility of developing a genetically diverse population in which different mutants will adapt to a patient’s stomach microenvironments, resulting in chronic infection or persistent colonization. The results of a study in Japan that analyzed the heterogeneity of *H. pylori* in human stomachs detected an extensive heterogeneity with more than 60% of patients having drug hetero-resistant strains in different locations of the stomach [41]. These findings also support the need for taking multiple samples from different parts of the stomach and detailed testing of samples for the detection of different phenotypes and genotypes in the same patient, resulting in potential eradication failure.

The resistance rates for both tested antibiotics had a significant rise detected during the study, especially during the COVID-19 pandemic emergence period. The increased trends of resistance among *H. pylori* are well known [8,9], which is why the recommendations in the Maastricht VI consensus suggest susceptibility testing even before prescribing first-line therapy [4]. There is a need for as much susceptibility testing as possible for *H. pylori.* This delivers effective therapy to the individual patient, lowers the rates of treatment failure and medical costs, monitors regional levels of resistance for empirical therapy options where testing is unavailable, and may lead to increased interest in *H. pylori* infection management strategies [5,42].

The present study has several limitations. First, it was a single center-based study from which the clinical biopsy samples were collected. On the other hand, this is a tertiary center for the Republic of Serbia, to which the majority of complicated patients are assigned. The only method for the determination of bacterial resistance was genetically by using a commercially available test (GenoType Helico DR). The test used is among commercially available molecular tests declared as most informative as it provides sensitivity to both fluoroquinolones and clarithromycin, with high sensitivity and specificity [43]. Despite other possible genetic determinants that are or may be linked with resistance to both fluoroquinolones and clarithromycin, the WGS studies revealed that these markers comprised in this test are sufficient for the reliable prediction of phenotypic resistance in *H. pylori* [32,43,44].

## 4. Materials and Methods

### 4.1. Patients

The adult patients were sampled and recruited from the Clinic for Gastroenterology of the University Clinical Center of Serbia, an Endoscopy Unit, in the period from April 2018 to December 2022. The patients originated from throughout Serbia, as they were sent from the Belgrade region, Clinical Centre Nis, and the regional hospitals in Novi Pazar, Uzice, Pancevo, and Sremska Mitrovica. Patients had previous positive noninvasive test results, which were conducted depending on the laboratory of the hospital where the patient originated (e.g., urea breath test, stool antigen test). Inclusion criteria were an age of over 18 years and symptoms related to diseases of the upper parts of the gastrointestinal tract (nausea, discomfort in the epigastrium, dyspepsia, weight loss, vomiting, etc.). Exclusion criteria were an age of less than 18 years, acute gastrointestinal bleeding, malignancy of the gastrointestinal tract and other localizations, allergy to the mentioned antibiotics, use of antibiotics in the last 2 months, pregnancy, and breastfeeding. Written informed consent was obtained from each participating patient before enrollment in the study. The research was conducted in accordance with the Declaration of Helsinki, and the protocol was approved by the Ethics Committee of the University Clinical Center of Serbia (788/11). During the esophagogastroduodenoscopy, biopsy tissue samples were taken with an endoscope according to the Updated Sydney System classification from the stomach’s antrum, angulus, and body for DNA extraction [45]. Biopsy specimens were placed in a sterile Eppendorf microtube (1.5 mL) with 1 mL of sterile normal saline and sent to the Institute for Microbiology and Immunology Medical Faculty University of Belgrade for molecular testing.

### 4.2. DNA Extraction and Molecular Testing by GenoType Helico DR Kit

For molecular conformation of *H. pylori* and resistance susceptibility testing to clarithromycin and fluoroquinolones in gastric biopsy samples, a GenoType Helico DR kit was used.

The biopsy samples were submitted for DNA extraction according to the GenoType Helico DR kit manufacturer’s suggestion using the QIAmp DNA Mini Kit (Qiagen, Benelux, The Netherlands). In brief, the tissue samples were completely lysed with ATL Buffer and Proteinase K at 56° C incubation in an Eppendorf ThermoStat Plus (Sigma-Aldrich, St. Louis, MO, USA). The elimination of contamination and enzyme inhibitors was conducted using spin columns, with further washing steps for the elimination of proteins and salts. The final elution of high-purity DNA was done using Tris–EDTA (pH 8.0) low-concentration elution buffer in aliquots of 50 µL. Isolated DNA samples were stored at −20 °C until further PCR amplification testing.

PCR reaction by multiplex amplification with biotinylated primers was performed for the identification of *H. pylori* and detection of clarithromycin (*rrl* gene) or fluoroquinolone (*gyrA* gene) resistance. According to the manufacturer’s instructions, the reaction mixture contained 5 μL of reaction buffer, 2.5 μL of MgCl_2_, 35 μL of biotinylated primers and nucleotide mixture, 0.4 μL of Taq polymerase, 2.5 μL of PCR grade water, and 5 μL of extracted DNA, all in a final volume of 45 μL. The PCR program was applied for biopsy samples: denaturation 1 cycle at 95 °C for 5 min, 10 cycles at 95 °C for 30 s and at 58 °C for 2 min, 25 cycles at 95 °C for 25 s, 53 °C for 40 s, and 70 °C for 40 s, with termination at 70 °C for 8 min.

The PCR products were submitted for hybridization with DNA strips. The reaction was performed using the TwinCubator (Hain Life Science, Nehren, Germany) system at a temperature of 45 °C, according to manufacturer protocol. Commercial strips contained 18 hybridization probes, two controls (conjugate and amplification), *H. pylori* detection, ten bands for quinolone, and five bands for clarithromycin sensitivity testing through the detection of wild-type (WT) and/or mutated alleles (MUT) for a respected antibiotic. For identification of clarithromycin resistance, mutations in the *23S rRNA* gene could be detected for A2142C, A2142G, and A2143G. Also, for fluoroquinolone resistance, mutations in *gyrA* gene N87K, D91N, D91G, and D91Y are designed for detection.

The interpretation of obtained results on DNA strips was also carried out using manufacturer instructions for combinations of detected or missing test bands on strips [13]. The presence of conjugation and amplification control bands must always appear, and the positivity of the HP (*H. pylori*) band with gyrA and 23S control bands in case of positive findings. The presence of WT and/or MUT bands or their absence indicated a sensitive or resistant resistance genotype of the tested *H. pylori* strain.

### 4.3. Statistical Analysis

Categorical data were compared using the Chi-square test. The level of statistical significance was specified as 0.05. The logistic regression model was used to examine the relationship between resistance and demographic variables. The odds ratios were estimated with 95% confidence intervals. Statistical analyses were performed using SPSS 20.0 (SPSS Inc., Chicago, IL, USA) statistical software.

## 5. Conclusions

The prevalence of *H. pylori* in symptomatic patients in Serbia corresponds to the global prevalence in adults. Resistance rates to fluoroquinolones and clarithromycin are evidently increasing, which is a significant challenge in the clinical management of *H. pylori* infection. These results point to the need to apply the latest recommendations by the Maastricht VI consensus for both susceptibility testing and guided therapy options. The present study is the first to provide overall genetically determined susceptibility results on *H. pylori* in Serbia. The GenoType HelicoDR kit is a useful diagnostic tool for antibiotic susceptibility testing, especially in cases of mixed *H. pylori* populations and multiple eradication failures. The perspective of personalized therapy based on molecular susceptibility testing is imposed as a possible future solution.

## Figures and Tables

**Table 1:** OR values estimates for clarithromycin and fluoroquinolone resistance according to age and gender.

Variables	Clarithromycin Resistant (n = 103)			Fluoroquinolone Resistance (n = 95)		
	% (n)	OR (95% CI)	*p*	% (n)	OR (95% CI)	*p*
Age						
≤50 (n = 72)	52.8 (38)	0.681 (0.366–1.268)	0.226	55.6 (40)	1.127 (0.615–2.066)	0.698
>50 (n = 104)	62.5 (65)	Ref		52.9 (55)	Ref	
Gender						
Male (n = 67)	46.3 (31)	0.446 (0.239–0.833)	0.011 *	49.3 (33)	0.732 (0.397–1.350)	0.318
Female (n = 109)	66.1 (72)	Ref		56.9 (62)	Ref	

* *p* < 0.05; OR—odds ratio; CI—confidence interval; Ref—reference.

**Table 2:** Detected mutations in fluoroquinolones and clarithromycin-resistant strains.

Antibiotic	Fluoroquinolones				Clarithromycin		
Gene	*gyrA 87*	*gyrA 91*			*rrl*		
Genotype	N87K (MUT)	D91N (MUT1)	D91G (MUT2)	D91Y (MUT3)	A2146G (MUT1)	A2146C (MUT2)	A2147G (MUT3)
Exchange	Asparagine/Lysine	Aspartate/Asparagine	Aspartate/Glycine	Aspartate/Tyrosine	AA/GA	AA/CA	AA/AG
∑(N/%)	26/27.4	23/24.2	19/20.0	14/14.7	25/24.3	5/4.8	81/78.6

MUT—mutation.

**Table 3:** Mixed genotype combinations detected among patients.

Fluoroquinolones Resistant			Clarithromycin Resistant		
Genotype	% of All Samples	% of Resistant Samples	Genotype	% of All Samples	% of Resistant Samples
WT 87 + MUT 87	6.3	3.4	WT + MUT (1–3)	9.1	15.5
WT 91 + MUT 91 (1–3)	7.4	4.0	>1 MUT	4.5	7.7
>1 MUT 91 (1–3)	22.1	11.9			
MUT 87 + MUT 91 (1–3)	8.4	4.5			
WT 87 + WT 91 + MUT 87 + MUT 91 (1–3)	2.1	1.1			

WT—wild type; MUT—mutation.

## Data Availability

Data will be made available on request.
